# Instability of (CTG)_n_•(CAG)_n _trinucleotide repeats and DNA synthesis

**DOI:** 10.1186/2045-3701-2-7

**Published:** 2012-02-27

**Authors:** Guoqi Liu, Michael Leffak

**Affiliations:** 1 Department of Biochemistry and Molecular Biology, Boonshoft School of Medicine, Wright State University, Dayton, OH 45435, USA

## Abstract

Expansion of (CTG)_n_•(CAG)_n _trinucleotide repeat (TNR) microsatellite sequences is the cause of more than a dozen human neurodegenerative diseases. (CTG)_n _and (CAG)_n _repeats form imperfectly base paired hairpins that tend to expand in vivo in a length-dependent manner. Yeast, mouse and human models confirm that (CTG)_n_•(CAG)_n _instability increases with repeat number, and implicate both DNA replication and DNA damage response mechanisms in (CTG)_n_•(CAG)_n _TNR expansion and contraction. Mutation and knockdown models that abrogate the expression of individual genes might also mask more subtle, cumulative effects of multiple additional pathways on (CTG)_n_•(CAG)_n _instability in whole animals. The identification of second site genetic modifiers may help to explain the variability of (CTG)_n_•(CAG)_n _TNR instability patterns between tissues and individuals, and offer opportunities for prognosis and treatment.

## Introduction

Expansion of (CTG)_n_•(CAG)_n _trinucleotide repeat (TNR) sequences at distinct chromosomal loci is the mutation common to multiple neurological diseases including myotonic dystrophy type 1 (DM1), Huntington disease (HD), Huntington disease-like 2 (HDL2), dentatorubral-pallidoluysian atrophy (DRPLA), spinal and bulbar muscular atrophy (SBMA), and several forms of spinocerebellar ataxia (SCA). The polyglutamine diseases HD, DRPLA, SBMA, and SCA1, 3, 6, 7, 17 result from increases of (CAG)n repeats in the coding (nontemplate) strand for mRNA synthesis of the cognate genes ((CAG)n in RNA) to produce mutant polyglutamine proteins with toxic gain-of-function [1]. In contrast, (CTG)n•(CAG)n expansion at the DMPK 3' UTR alters the chromatin structure of the region, downregulates transcription of the locus and, as at the JPH3 gene produce poly-(CUG) pre-mRNAs respectively in DM1 and HDL2 patients that sequester the MBNL (CUG) binding proteins, leading to *trans*-dominant interference with the normal splicing of multiple RNAs. Finally, bidirectional transcription at the SCA8 locus can result in expression of both a polyglutamine protein and a (CUG)n expansion transcript, which may represent a toxic gain-of-function at both the protein and RNA levels.

**Figure 1:**
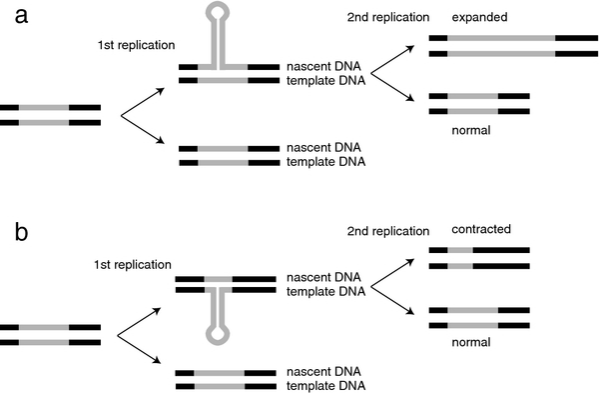
**Hairpin-induced trinucleotide repeat instability**. The TNR is indicated by gray lines, flanking DNA by black lines. (a) Nascent-strand hairpin formation results in over-replication of a segment of the TNR in one chromatid. A second round of replication of the hairpin strand fixes the expanded allele in the genome. (b) Template-strand hairpin formation results in under-replication of a segment of the TNR in one chromatid. A second round of replication of the nonhairpin strand fixes the contracted allele in the genome. (Reprinted from [7] with permission)

Trinucleotide repeat expansion requires DNA synthesis, either during DNA replication or repair. The effects of replication origin proximity, replication polarity, and replication inhibition support replication-based models of TNR instability in mitotic cells [2-9]. Hairpin formation by DNA polymerase slippage is a likely mechanism for changes in TNR repeat length [10-12]. Hairpin structure formation by DNA polymerase slippage at (CTG)_n_•(CAG)_n _sequences has been well documented in vitro [13,14] and can result in either insertion or deletion mutations. However, hairpins have also been postulated to arise during replication fork reversal and postreplication repair [2,15,16], Okazaki fragment maturation [17-19], base excision repair [20], nucleotide excision repair [21-26] or repair of structures induced by R-loop formation during transcription [25,27]. Current models of (CTG)_n_•(CAG)_n _instability during replication or repair envision that hairpin formation on the newly synthesized DNA strand leads to TNR expansion if the hairpin is sufficiently long-lived to serve as template in a subsequent round of replication. Conversely, stable hairpin formation in the leading or lagging template strand would lead to contraction of the repeat in the next round of replication (Figure 1).

The salient observation that TNR instability in humans and mice can occur in postmitotic cells argues that repair mechanisms, instead of replication origin-dependent mitotic DNA replication, are involved in TNR instability in these tissues [2,5,28-30]. In this vein, it has been proposed that the process of transcription stimulates TNR instability due to the formation of hairpin or other non-B DNA structures in the single stranded nontemplate DNA, or in the template strand upon RNA displacement. These structures may be targets for DNA repair processes such as transcription-coupled repair, nucleotide excision repair, mismatch repair, or double-stranded DNA break repair [24,27,31].

Following extensive linkage analysis in myotonic dystrophy families [32-34], in 1992 several laboratories reported that expansion of the (CTG)_n_•(CAG)_n _repeat region in the 3' untranslated region of the dystrophia myotonica protein kinase gene was highly correlated with the occurrence of congenital DM [35-37]. Strong correlations also exist between (CTG)_n_•(CAG)_n _repeat length and the occurrence of Huntington disease [38,39], although second site modifier genes and epigenetic mechanisms play a significant role in the appearance of HD symptoms. In general, unaffected individuals display fewer than 30 (CTG)_n_•(CAG)_n _repeats at the DM1 or HD locus. Trinucleotide repeat (TNR) tracts in the range of 30-40 repeats are termed premutation alleles (DM1) or intermediate alleles of incomplete penetrance (HD), while TNRs of 42 or more repeats have been associated with complete penetrance of HD [40] and increased expansion frequency during intergenerational transfer or somatic development in DM1 families [2]. The phenomenon of 'genetic anticipation' is a hallmark of the (CTG)_n_•(CAG)_n _TNR instability disorders, in which an increase in the number of microsatellite repeats is correlated with an earlier age of onset and heightened severity of the disease in successive generations. Genetic anticipation reflects the bias towards expansion over contraction of long (CTG)_n_•(CAG)_n _tracts, and may be explained by the greater tendency of extended repeats to adopt non-B form DNA structures prone to progressive expansion.

(CTG)_n_•(CAG)_n _expansion can have pathological effects on local chromatin structure and gene expression, as well as dominant negative effects on RNA metabolism and protein function [41-44]. This review will focus primarily on the structure and instability of (CTG)n•(CAG)_n _trinucleotide repeat sequences in eucaryotic cells. For further background on the metabolism of (CTG)_n_•(CAG)_n _sequences in bacterial cells, the reader is referred to several excellent research articles and reviews [5,6,45-51].

### (CTG)_n_•(CAG)_n _hairpins in vitro

NMR, melting and chemical modification analyses confirm that both (CTG)_n _and (CAG)_n _oligonucleotides as short as 6-10 repeats can form stable hairpin structures with mismatched base pairs [21,52-55]. Although short (CTG)_n _or (CAG)_n _hairpins represent stable structures, they rapidly convert to duplex DNA in the presence of the complementary oligonucleotide through loop-loop and stem-stem interactions without prior denaturation [52]. Considered with the greater thermodynamic stability of duplex vs. cruciform DNA, and the inhibition of cruciform formation by single base mismatches [56,57], these observations suggest that unless otherwise stabilized e.g. by protein binding or high negative superhelicity [2,58], apposed hairpin structures formed in vivo by transcription or replication fork regression to "chicken foot" structures would resolve to duplex DNA, and disfavor TNR expansions. Notably, (CTG)_25 _or (CAG)_25 _hairpins form duplex DNA approximately 5-fold more slowly than (CTG)_10 _or (CAG)_10 _hairpins in the presence of their complementary strands, despite similar thermal stabilities of the hairpins [55]. Thus, longer hairpins may also have longer half-lives in vivo.

Compared to nonrepetitive palindromic sequences, which would require half of the palindrome to become single stranded prior to hairpin formation, the free energy required to nucleate short hairpin formation may be provided by negative superhelicity [2,58]. The greater number of hairpin configurations in a repetitive TNR would be expected to increase the entropy of hairpin formation and decrease the free energy. Thus, in addition to the slower dissolution of longer hairpins by their complementary sequences, hairpin formation may occur more rapidly in longer repeats, competing against SSB binding.

To assess the mechanism of hairpin instability, Panigrahi et al. used in vitro replication of (CTG)_79_•(CAG)_79 _repeats driven by the SV40 T antigen (T-ag) helicase. The remaining enzymatic machinery of DNA synthesis is endogenous to the host cell. Plasmids in which the (CAG)_n _sequence was the lagging strand template showed an expansion bias, while plasmids containing the opposite TNR replication orientation ((CTG)_n _in the lagging strand template) displayed a preference for contraction [59]. To the extent that the replication fork driven by the strong T-ag helicase mimics the activity and interactions of the cellular Cdc45/MCM2-7/GINS replicative helicase [60,61] with replication fork stabilizing proteins, the effect of TNR orientation relative to the replication origin on (CTG)_n_•(CAG)_n _stability imply that unstable TNR structures can be processed to expansions or contractions depending on DNA replication polarity.

The question of why a contraction bias is observed in rapidly dividing eucaryotic cells [16] was addressed by Delagoutte et al. using a primer extension model of (CTG)_n_•(CAG)_n _replication, in which replication by T4 DNA polymerase through short (CAG)_n _or (CTG)_n _TNRs was inhibited relative to polymerization through non-structure forming repeats. (CAG)_n _repeats blocked replication more efficiently than (CTG)_n _repeats, and this difference was eliminated by the addition of E. coli or T4 single strand binding SSB proteins [62]. Based on the preferential binding of SSB to lagging strand template DNA and the more efficient blockage of polymerization by the (CAG)n vs. (CTG)n template, the authors proposed a 'template-push' model in which the contraction bias of TNRs with (CTG)n in the lagging strand template is not the result of lagging strand structure formation while single stranded, but is the result of extrusion of the leading strand (CAG)_n _template and replication across the abasic bottom of the hairpin in order to maintain contact between DNA polymerase and replicative helicase [62]. Alternatively, transient release of the leading strand hairpin template from the stalled polymerase could allow hairpin slippage or migration in the 5' -- > 3' direction away from the replication fork [13,63,64], and reestablishment of a functional primer-template junction.

Annealing of single stranded plasmid DNA to complementary strands containing excess (CTG)_n _or (CAG)_n _sequences, which formed hairpins as large as 25 repeats, yielded products that showed accurate repair in human cell extracts [56,65-68]. The requirement for PCNA, and the nick-dependence of accurate repair suggested that mismatch repair proteins (MMR) that function during replication in vivo might play a role in hairpin resolution in vitro. Indeed, in one study, the repair of short (CTG)_1-3 _slip out structures was reported to increase with increasing concentrations of MutSβ (Msh2/Msh3) in cell extracts [69]. Nevertheless, in contrast to the apparent requirement for MMR proteins for (CTG)_n_•(CAG)_n _expansions in transgenic mice [70-72], MMR proteins, or the nucleotide excision repair (NER) protein XPG, were not essential for repair of longer (CTG)_20-25 _hairpins in cell extracts [66,67,69]. It remains possible that there are alternative pathways for hairpin removal. Additionally, the formation of stable hairpins in advance in these assays may have bypassed the contribution of MMR, NER or other pathways to hairpin repair.

### Yeast models of (CTG)_n_•(CAG)_n _instability

Numerous genetic analyses have been performed in *S. cerevisiae *to characterize the effect of (CTG)_n_•(CAG)_n _sequences on DNA replication and chromosome fragility, and to identify proteins that affect (CTG)_n_•(CAG)_n _trinucleotide repeat stability. Between these studies there are some disparities that are likely due to differences in the repeat sequence ((CTG)_n_•(CAG)_n_, (GAA)_n_•(TTC)_n_, (CGG)_n_•(CCG)_n_), its environment (plasmid vs. chromosome; leading vs. lagging strand replication polarity), number of repeats in the microsatellite tract, genetic background, and the sensitivity of the assay to small changes in repeat length [73-82]. Thus the frequency of expansions was approximately 500-fold greater when (CTG)_25 _was in the lagging strand template than when (CAG)_25 _was in the lagging strand template [17]. While the frequencies of (CTG)_25 _or (CAG)_25 _expansions, and (CTG) _50 _or (CAG)_50 _contractions, were unaffected in *msh2 *mutants [17,83], (CTG)_13 _expansion was stimulated by mutation of postreplication repair genes *rad18 *(hRAD18, binding partner of hUBE2A/B), *rad5 *(hSMARCA3), and PRR-specific alleles of *pol30 *(hPCNA) [74].

When analyzed by two-dimensional gel electrophoresis (CTG)_80_•(CAG)_80 _sequences showed only modest effects on replication fork progress, irrespective of replication polarity. In contrast, (CGG)_40_•(CCG)_40 _repeats imposed strong blocks to fork progression [75]. Surprisingly, in an assay that used reversion to 5-fluoroorotic acid resistance (FOA^R^) to quantitate TNR expansions, comparable rates of repeat instability were found for (CGG)_25 _or (CTG)_25 _(lagging strand template) TNRs. In the presence of the *rfc1-1 *mutation, which blocks PCNA loading and lagging strand Okazaki fragment synthesis, the expansion rates of (CGG)_25 _and (CTG)_25 _increased ~40-50 fold and ~2-3 fold respectively. One interpretation of this result is that inhibition of lagging strand synthesis can promote expansions in the leading strand nascent DNA. However, since (CGG)_n _and (CTG)_n _repeats in the lagging strand template characteristically show a strong bias towards contraction, this assay may not have revealed the full relationship between replication stalling and TNR instability. In a similar assay, expansion of (CAG)_25_•(CTG)_25 _was increased ~100 fold when (CAG) was in the lagging vs. leading strand template, and a *ra27*Δ mutant in the Okazaki flap endonuclease (hFEN-1) enhanced (CTG)_n_•(CAG) _n _expansion an additional 100 fold, irrespective of replication orientation [84].

Bhattacharya and Lahue [85] reported that (CTG)_13 _(lagging strand template) expansion was markedly (~40 fold) increased in *srs2 *helicase mutants, while (CTG)_25 _expansion was increased ~5 fold in the same cells, and these rates were minimally affected by mutation of the RecQ helicase *sgs1 *or either of the homologous recombination proteins *rad51 *or *rad52*, arguing against unequal sister chromatid exchange as a mechanism of expansion, consistent with the absence of exchange of markers flanking expanded alleles in human patients [86,87]. These results differed from those of Kerrest et al. who reported that the fragility of yeast artificial chromosomes (YACs) containing longer (CTG)_70_•(CAG)_70 _TNRs, which are above the expansion threshold, increased significantly in *sgs1*Δ or *srs2*Δ helicase mutants. Deletion of the homologous recombination protein genes mitigated the effect of the *srs2*Δ mutation in either orientation, and decreased the effect of the *sgs1*Δ mutation in the (CTG)_70 _orientation, but exacerbated the effect of the *sgs1*Δ mutation in the (CAG)_70 _orientation [15]. A simple relationship between YAC fragility, TNR length and replication polarity was difficult to ascertain for these mutants, leading the authors to suggest that multiple pathways coexist that involve Srs2, Sgs1, Rad51 and Rad52 in the repair of replication fork damage due to hairpin forming sequences of different lengths and orientations.

In a screen for mutants that affected (CTG)_n_•(CAG)_n _instability, disruption of the replication fork stabilization complex protein genes Mrc1, Tof1 or Csm3 selectively enhanced contractions of a (*CAG)_20_*-URA3 (lagging strand template) reporter, independent of replication checkpoint and DNA damage checkpoint factors [76], and control experiments demonstrated that mutation of the same fork stabilization complex proteins did not affect the stability of a non-structure forming (CTA)_n _repeat. In contrast, mutation of the fork stabilization complex proteins or the DNA damage checkpoint proteins Ddc1, Rad9, Rad17, Mec1, Ddc2, Rad24, Mec3, Rad53 or Chk1 led to increased expansion of a (*CAG)_13_*-URA3 reporter. These results suggest that Mrc1, Tof1 and Csm3 may maintain TNR length through coupling of the DNA polymerase and replicative helicase to prevent the formation of hairpins, whereas the DNA damage checkpoint is involved in stabilization of the replisome after the formation of hairpin structures.

Assays using longer TNRs (85-155 repeats) in *mrc1, rad9, mec1, ddc2, rad17, rad24, chk1, or rad53 *mutant strains found elevated chromosome breakage due to expanded (CAG)_n_•(CTG)_n _tracts and increased instability (primarily contractions) of a (CAG)_n _(lagging strand template) reporter [77,78]. The inherently greater instability of long (CTG)_n_•(CAG)_n _repeats in wild type strains may have masked the effects of some checkpoint mutants, nevertheless, these studies indicate that distinct protein complexes respond to forms of DNA replicative stress that differ in size or geometry, and underscore the correlation between noncanonical (CTG)_n_•(CAG)_n _structures, checkpoint activation and chromosome breakage [88,89]. Indeed, Sundararajan et al. have recently shown that long (CTG)_n_•(CAG)_n _tracts induce chromosomal double strand breaks in yeast, and that the Mre11/Rad50/Xrs2 complex is necessary for blocking chromosome fragility and inhibiting (CAG)_70 _TNR instability (expansion and contraction) by both homologous recombination and NHEJ pathways [90].

The homologous recombination protein Rad52 was also required to protect the (*CAG)_70_*-URA3 reporter from length instability and chromosome breakage in the presence of mutations in the alternative clamp loader Ctf18-Dcc1-Ctf8-RFC (Ctf18-RFC) [91]. Previously thought to promote PCNA loading and unloading during replication fork navigation through sister chromatid cohesion (SCC) complexes [92,93], Gellon et al. showed that Ctf18-RFC is required for TNR stability independent of its role in SCC, in parallel to a pathway involving the Mrc1 protein which couples the leading strand polymerase ε and the replicative helicase at the replication fork, and acts in signaling during the intra-S phase checkpoint and the DNA damage response [94-96]. On the replication fork lagging strand, Ctf4 collaborates with MCM10 to link DNA polymerase α to the MCM2-7 helicase [97-99]. Like the *mrc1 *mutant, a *ctf4 *deletion mutant is associated with chromosomal instability, *ctf4 rad52 *double mutants grow poorly and produce a high percentage of inviable cells [100], and *ctf4 mrc1 *mutants are inviable [101].

Taken together these studies in yeast suggest a cellular fail-safe strategy of overlapping pathways to (i) prevent the formation of stable hairpin structures by maintaining the rate of replisome movement and coupling of leading and lagging strand polymerases to the replicative helicase, (ii) restore hairpin structures to duplex DNA by repair helicases, and (iii) recruit postreplication repair machinery to excise hairpins.

### Mouse models of (CTG)n•(CAG)n instability

Murine models of several trinucleotide expansion diseases including Huntington disease, myotonic dystrophy type 1, Fragile X syndrome, and Friedrich's ataxia have been generated by random integration of pathological length repeat tracts or knock-in at homologous genetic sites, and have reproduced many, though not all, phenotypes of the associated disease. These models typically show tissue-specific, expansion-biased patterns of instability similar to those in humans, including expansions in germ cells, early embryos and adults. Although intergenerational (CTG)_n_•(CAG)_n _expansion is typically smaller in transgenic mice than humans [16,102], some recent studies have reported relatively large expansions during parent-to-offspring transmission [103,104]. Among the *cis*-acting modulators of TNR instability in murine systems are the sequence and length of the TNR [105], the presence of human flanking DNA [27,106-108], the chromosomal integration site [109,110], chromatin structure [41,43,111-115], and replication polarity [4].

Relevant to studies of the relationship between the DNA replication and (CTG)_n_•(CAG)_n _stability is a comparison of origin activity at the DMPK locus in human cells and transgenic mice [4]. In this work, two origins were mapped upstream and downstream of the DMPK (CTG)_n_•(CAG)_n _repeat in both control and DM1 human fibroblasts. Transgenic mice bearing a single copy of a ~45-kb genomic region of the expanded DM1 locus containing (CTG) _> 300_•(CAG) _> 300 _repeats showed high levels of intergenerational and somatic repeat instability [116]. The transcriptional activity of the DM1 locus and tissue-specific patterns of instability were similar to those of DM1 individuals. Unlike in humans, however, when origin activity (abundance of nascent DNA) was quantitated over the ~45-kb human DM1 transgene from pancreatic cells of mice bearing either > 300 (DM328) or 20 (DM20) [117] repeats, neither the upstream nor the downstream origin was active in DM20 mice, and only the upstream origin was inactive in DM328 mice, [4]., Thus, the nuclear environment of the transgene may also modulate its replication origin activity and downstream effects on TNR stability.

Conversely, pathological length (CTG)_n_•(CAG)_n _transgenes could induce local heterochromatinization and position effect variegation (PEV) upon integration. With overexpression of the heterochromatin organizing protein HP1β, PEV increased only in transgenes containing the TNRs [43]. These data suggest that the integration site and the transgene may each effect biological pressure for or against integration of a DNA fragment at a particular genomic site. While the influences of chromosome environment are manifold, *cis*-effects of the integration site on TNR instability are generally diminished with increasing length of the microsatellite repeat tract and the human flanking DNA [27,107].

Transcription is a possible *cis*-acting modifier that could lead to tissue-specific TNR instability, although the constitutive expression of the associated disease genes in humans argues against this model, and no correlation was observed between instability and stable mRNA levels in DM1 [118], HD [119] or SCA7 (CTG)_92_•(CAG)_92_) transgenic mice [115]. Nevertheless, secondary attributes of transcription, e.g. DNA supercoiling, histone modification, DNA repair induced by the formation of RNA-DNA hybrid loops [25,120,121] may indirectly account for the activation of ATR or ATM pathways [122-124], and transcription induced (CTG)_n_•(CAG)_n _contraction has been reported in non-murine systems [26,125-129].

Specific *trans*-acting factors that have been implicated in (CTG)_n_•(CAG)_n _instability based on crosses between transgenic TNR mice and mice defective in DNA mismatch repair or base excision repair [20,71,72,102,130,131]. In bacteria, MMR relies primarily on three protein complexes MutS, MutL and MutH [132]. The MutS dimer recognizes the mismatch and enlists a MutL dimer that then recruits the MutH endonuclease to initiate nick-directed repair. In eukaryotes there are at least six homologs to the MutS and MutL proteins [133,134]. The major MutS homologue is MSH2, which can heterodimerize with either MSH6 to form MutSα which binds to single base mismatches, or with MSH3 to form MutSβ that recognizes short insertion/deletion loops. The PMS2/MLH1 (MutLα) heterodimer interacts with mismatches recognized by MutSα or MutSβ to trigger downstream excision and resynthesis reactions.

The murine mismatch repair genes MSH2 and MSH3 are essential for germinal and somatic expansion, and PMS2 is required for somatic instability, of long (> 84 repeat) (CTG)_n_•(CAG)_n _TNRs in transgenic mice [16,70-72,102,116,130,131,135,136]. In vitro, MutSβ binding to (CAG)_n _hairpins nominally reduced its ATPase activity, suggesting that MutSβ might mask hairpin structures from repair [137,138]. However, a later study reported that the ATPase activity of MutSβ bound to (CAG)_n _hairpins was similar to that of the enzyme bound to nonhairpin duplex DNA, and that (CAG)_n _hairpin binding of MutSβ did not change its catalytic efficiency (kcat/Km) [139]. Thus, the precise mechanism by which the MMR system is involved in TNR instability in vivo remains unresolved.

When transgenic mice carrying the HD (CAG)_n _repeat were crossed with mice lacking the base excision repair (BER) glycosylase OGG1 (7,8-dihydro-8-oxoguanine DNA glycosylase), which is responsible for the removal of 7,8-dihydro-8-oxoguanine (8-oxoG) (the most common oxidized base in DNA), age-dependent somatic expansion was largely suppressed [20]. In an in vitro model of base excision repair, incision of 8-oxo-G within a (CAG)_n _tract by APE1 and extension by the major BER polymerase, polβ, resulted in expansion of the repeat tract [20,140], leading to the hypothesis of a toxic oxidation cycle in which hairpin loops form during long-patch repair of bases damaged by reactive oxygen species. The hairpin may be protected from the endonuclease activity of FEN1 by MutSβ [141], or FEN1 may promote the ligation of hairpin-containing flaps [140]. Repeated cycles of oxidation, repair and expansion would promote progressive age-dependent expansion. The presence of additional glycosylases that can act on 8-oxo-G (and other oxidized bases) implies a unique function for OGG1 in (CAG)_n _expansion during BER, which is not yet understood [142]. Similar experiments in which DM328 mice ((CTG) _> 300_•(CAG) _> 300 _repeats) were crossed with mice deficient in Rad52, Rad54 or DNA-PKcs showed little effect on TNR stability, arguing that homologous recombination or nonhomologous end joining are not involved in expansion or contraction [135].

A difference between yeast and murine systems is the reported absence of an effect of Fen1 loss on TNR instability in DM1 knock-in transgenic mice [143]. Fen1 knockdown (80-90%) did not affect the stability of the HD (CAG)_27_•(CTG)_27 _repeats in human cells, but Fen1 haploinsufficiency did induce expansion of transgenic (CAG)_120_•(CTG)_120 _repeats [144]. Constitutively low levels of Fen1 have also been implicated in inducing (CAG)_n_•(CTG)_n _instability in the striatum vs. cerebellum of HD mice [145].

The similarity of tissue-specific patterns of somatic mosaicism in multiple mouse models also suggest the influence of additional tissue-specific factors affecting TNR stability [104,110,118,119,146]. Further, it has been proposed that sex-specific trans-acting factors are responsible for differences in intergenerational instability between murine and human systems [103]. A recent genomic study compared the phenotype of tissue-specific patterns of (CAG)_n_•(CTG)_n _instability of *Hdh^Q111 ^*(HD homolog) transgenic mice with microarray analysis of gene expression [147]. The collective expression signature of a group of 150 genes was highly correlated with tissue instability, although no single gene expression pattern was absolutely predictive of instability. Instability indices were highest in nondividing striatum (highly affected in HD) and liver cells, and lowest in testis and umbilical cord. Comparison of *Hdh*^*Q*111/111 ^and *Hdh*^+/+ ^littermates showed that the instability of normal or mutant striata was significantly higher than the instability index of cerebellum. The authors concluded that mutant and wild type striata have similar tendencies towards TNR expansion, but the HD (CAG)_n_•(CTG) _n _microsatellite does not expand in normal striatum because it is not of sufficient length to be susceptible to additional processes involved in expansion. Possibly, transient or short-lived fluctuations in protein function or DNA structure occur frequently in specific tissues to increase the susceptibility of long TNRs to expansion.

In contrast to previous reports that MMR and BER proteins contribute to (CTG)_n_•(CAG)_n _expansion in mice, expression levels of DNA repair genes including MSH2, MSH3, and OGG1 did not correlate with the tissue specificity of somatic instability [147]. To address the caveat that steady state RNA levels may not reflect changes in protein abundance, the authors confirmed by immunoblot that Cbp and MSH2 protein protein levels were indistinguishable in *Hdh^+/+ ^*and *Hdh^Q111/+ ^*mice. However, further inspection of the data revealed that 63 of 74 genes whose downregulation showed weak-to-medium range Pearson coefficient correlation to TNR instability are involved in DNA metabolism. The authors concluded that pathways including cell cycle, metabolism and neurotransmission act in combination to generate tissue-specific patterns of instability, and that multiple tissue factors reflect the level of somatic instability in different tissues. Components of any of these pathways may represent second site genetic modifiers that contribute to the tissue- and cell type-specific variation of (CTG)_n_•(CAG)_n _TNR instability observed in mice and humans.

### Human models of (CTG)n•(CAG)n instability

During human and mouse development, DM1 tracts tend to expand in premeiotic spermatogonia, and large alleles subsequently contract during later stages of spermatogenesis and early in male development. In females, large expansions can be observed in nondividing oocytes, and full mutations are inherited almost exclusively from the mother. Thus, it has been proposed that two mechanisms of instability apply to (CTG)_n_•(CAG)_n _repeats: as in oocytes, expansions occur by DNA repair, while contractions characteristic of male development are the result of DNA replication [142]. In both male and female DM1 patients (and transgenic mouse models), (CTG)_n_•(CAG)_n _TNR tracts also show a significant level of somatic instability that increases with age in a tissue-specific manner. In possible support of a dual mechanism model for expansions and contractions, DM328 transgenic mice made deficient in DNA ligase I displayed reduced (CTG)_n_•(CAG)_n _instability upon maternal transmission, but showed no effect on paternal transmission or somatic instability [148]. Although studies such as these are valuable in identifying candidate genes affecting (CTG)_n_•(CAG)_n _instability, non-human model systems arguably do not recapitulate all aspects of microsatellite expansion disease in human cells due to differences in chromatin structure, cell division and DNA replication rates, and cell type. Hence, several investigators have turned to analyses of patient-derived cells, human embryonic stem (hES) cells [149-151], and other human cell model systems [7].

Recent PCR and immunoblot studies reported that MMR (MSH2, MSH3, MSH6) gene expression and protein levels of VUB03_DM1 and VUB19_DM1 hES cells were as high as in MMR proficient HeLa cells and stable during culture in the undifferentiated state, when (CTG)_n_•(CAG)_n _repeat length increased significantly [151,152]. Following differentiation to osteoblast progenitor-like cells, sharp decreases in MSH2, MSH3 and MSH6 levels were correlated with stabilization of (CTG)_n_•(CAG)_n _repeat lengths of the VUB03_DM1 and VUB19_DM1 hES cells [152]. These results imply that either the reduction in MMR protein expression, the decrease in cell proliferation during hES cell differentiation, or other *trans*-acting factors, may be related to (CTG)_n_•(CAG)_n _TNR stabilization.

In a human fibrosarcoma model that scores contraction of an intronic (CTG)_95_•(CAG)_95 _repeat by cell survival under HPRT+ selection (HAT medium), ~25-fold induction of transcription of randomly integrated HPRT cassettes increased contraction ~15-fold, to roughly 0.001% of cells. Transcription-induced contraction frequencies accumulated at the same rate in proliferating and confluent cells that differed by 10-fold in rates of cell division. siRNA knockdown of proteins involved in mismatch repair (MSH2, MSH3), and transcription-coupled nucleotide excision repair (CSB, ERCC1, XPA, XPG, TFIIS, BRCA1, BARD1) decreased the frequency of contractions 2- to 3-fold [25,26,126,153], indicating that these pathways likely play a role in TNR expansion in postmitotic cells. Supporting a role for BER in somatic alteration of (CAG)_n_•(CTG)_n _repeat length, tissue-specific decreases of (CAG)_n_•(CTG)_n _instability in the striatum, cerebral cortex and hippocampus of *Xpa*^-/- ^SCA1 mice have recently been reported [154]. Nevertheless, a parsimonious mechanism for transcription-induced instability that addresses both supporting and conflicting evidence is not yet available [25].

The effect of DNA replication on the stability of (CTG)_n_•(CAG)_n _sequences has been studied in SV40 origin plasmids replicating in COS-1 cells [155] or T-ag supplemented HeLa cell extracts [59]. While these systems do not duplicate the chromatin structure of genomic DNA, and replication does not utilize components of the ORC-dependent replisome that interact with the cellular DNA damage signaling and repair machinery, (CTG)_n_•(CAG)_n _instability in these plasmids was sensitive to TNR length, leading/lagging strand replication polarity, and distance to the viral replication origin. Hence, strong evidence supports both replication-dependent and replication-independent mechanisms of (CTG)_n_•(CAG)_n _instability.

A pharmacological approach to reducing the length of (CTG)_n_•(CAG)_n _TNRs was used by Hashem et al. in lymphoblast cell lines derived from DM1 patients (CTG)_~770_•(CAG)_~770 _repeats) [156]. Short term treatment with several DNA damaging drugs (ethylmethanesulfonate, mitomycin C, mitoxantrone, doxorubicin) led to the accumulation of smaller (CTG)_n_•(CAG)_n _repeat alleles in the cell population, often to fewer than 100 repeats. The rate of shift in the population profile indicated that the effects of the drugs were on the TNRs directly, rather than through mitotic selection. This result is significant given the tendency of the DM1 (CTG)_n_•(CAG)_n _repeats to expand rather than contract in patients and in culture. A similar study by Yang et al. showed that the replication inhibitors aphidicolin (which inhibits both leading and lagging strand DNA polymerases [157]) and emetine (which selectively blocks lagging strand Okazaki fragment synthesis [158]), but not mimosine (which induces DNA double strand breaks and arrests cells in late G1 phase [159,160]), increased the rate of (CTG)_n _expansion in DM fibroblast cells. In these experiments only the expanded DM1 allele ((CTG)_~220_•(CAG)_~220_) was altered, leaving the normal allele, (CTG)_12_•(CAG)_12 _unaffected. Aphidicolin and emetine enhanced the magnitude of short expansions in almost 100% of cells approximately three-fold, while up to 25% of cells gained more than 120 repeats. Likewise, in kidney cells from *Dmt*-D transgenic mice carrying (CTG)_160_•(CAG)_160 _repeats, Gomes-Pereira and Monckton [161] showed that prolonged exposure to the nucleoside analog and chain elongation inhibitor cytosine arabinoside, the intercalating mutagen ethidium bromide, the DNA methylation inhibitor 5-azacytidine, and aspirin, reduced the rate of repeat expansion, while exposure to caffeine, which uncouples DNA replication and repair from cell cycle checkpoints, increased the rate of expansion.

An alternative approach to the study of (CTG)_n_•(CAG)_n _instability was taken by Liu et al., who constructed a clonal HeLa cell line containing a single FLP recombinase target site into which (CTG)_n_•(CAG)_n _repeats of various lengths were integrated alongside the human c-myc replication origin in either replication orientation at the same ectopic chromosomal site [7]. In these HeLa/c-myc:(CTG)_n_•(CAG)_n _cell lines, the (CTG)_n_•(CAG)_n _tracts displayed time-, replication polarity-, and repeat length-dependence of instability. Moreover, treatment of these cells with emetine, FEN1 siRNA or low dose aphidicolin rapidly (< 10 population doublings) and efficiently induced instability of the premutation length (CTG)_45_•(CAG) _45 _and disease-related (CTG)_102_•(CAG)_102 _TNRs, but not normal length (CTG)_12_•(CAG)_12 _TNRs. For all three treatments (low dose aphidicolin, emetine, siRNA) there was a bias towards contraction when (CTG)_102 _was in the lagging strand template, and towards expansion when (CAG)_102 _was in the lagging strand template. The presence of (CAG)_n _in the lagging strand template is the same replication polarity that has generated (CTG)_n_•(CAG)_n _expansions in all other model systems [5,162]. Additional RNAi experiments using these cells (GL, ML, submitted) have confirmed the results of yeast studies in which mutation of Tof1 (human Timeless), Csm3 (human Tipin), or Mrc1 (human Claspin) dramatically increased similar patterns of (CTG)_n_•(CAG)_n _instability.

In general, the treatment of cultured human or transgenic mouse cells with DNA damaging drugs or replication inhibitors demonstrates that environmental agents can modulate (CTG)_n_•(CAG)_n _microsatellite instability, and that agents that cause acute DNA damage or repair are at least three orders of magnitude more efficient at inducing TNR instability than transcription-induced destabilization [153], although a unifying mechanism for explaining the observed changes has not emerged.

Towards clarifying the relationship between replication and expansion of the DMPK (CTG)_n_•(CAG)_n _TNR, Cleary et al. analyzed origin activity across the DMPK locus in age-, tissue- and sex-matched human control and DM1 fibroblasts [4]. These experiments revealed two replication origins, upstream and downstream of the DMPK (CTG)_n_•(CAG)_n _repeats in both control and DM1 cells. Our laboratory has independently confirmed the presence of origins upstream and downstream of the DMPK (CTG)_n_•(CAG)_n _repeats in matched DM1 and non-DM1 cells. The upstream origin coincides with that found by Cleary et al., while the downstream origin is approximately 2 kb closer to the TNR (GL, ML, submitted). Cleary et al. also mapped the activity of these origins in transgenic mice containing the ~45 kb DMPK locus from control (DM20, (CTG)_~20_•(CAG)_~20_) or DM1 (DM328, (CTG) _> 300_•(CAG) _> 300_) and found that only the origin downstream of the expanded (CTG) _> 300_•(CAG) _> 300 _TNR was active. It is thus possible that the downstream origin (which positions (CAG)_n _in the lagging strand template) is responsible for replication and expansion of the TNR in the transgenic DM328 cells. However, extension of this interpretation to human cells or other chromosomal environments is clouded by the observations that, in contrast to the transgenes, both upstream and downstream origins were equally active in human control and DM1 fibroblasts, while integration of the nonexpanded control (DM20) DMPK locus resulted in inactivation of both upstream and downstream origins in the transgenic mice.

**Figure 2:**
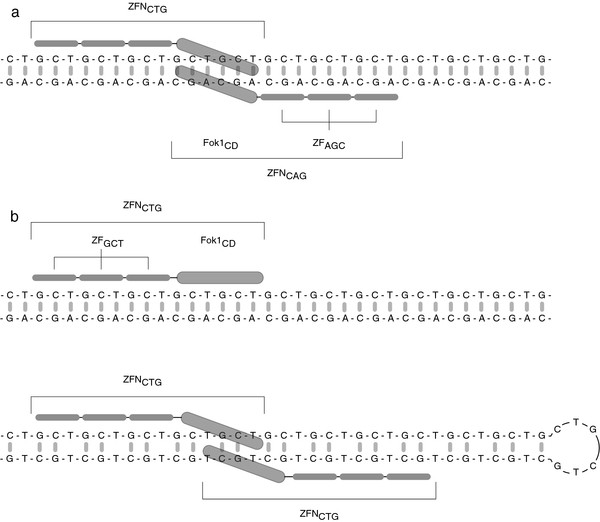
**Predicted modes of ZFN binding**. (a) Binding of a ZFN_CTG _and ZFN_CAG _heterodimer capable of cleaving heteroduplex DNA. FokI CD, FokI catalytic domain; ZFP_GCT_, (GCT)-recognition zinc finger protein; ZFP_AGC_, (AGC)-recognition zinc finger protein. (b) Predicted modes of ZFN_CTG _monomer binding to heteroduplex DNA (upper) or homodimeric ZFN_CTG _capable of cleaving (CTG) hairpin DNA (lower). (Reprinted from [7] with permission)

As discussed above, (CTG)_n_•(CAG)_n _instability is believed to result from the formation of hairpins in template strand DNA leading to contractions, or in newly synthesized DNA leading to expansions. Nevertheless, direct proof of hairpin formation in vivo has been lacking. To test for the presence of hairpins in vivo, synthetic zinc finger proteins (ZFPs) were engineered that specifically recognize either the (CTG)_n _strand or (CAG)_n _strand of the DMPK TNR, and fused to the Fok1 nuclease catalytic domain. The resulting zinc finger nucleases (ZFNs) dimerize only after zinc finger binding to their respective DNA substrate, which activates the nuclease catalytic domains. As diagrammed in Figure 2, heterodimerization of ZFN_CTG _and ZFN_CAG _is required to cleave Watson-Crick duplex DNA. However, hairpin DNA presents the same sequence ((CTG)_n _or (CAG)_n_) on both legs of the stem, and can be cleaved by a ZFN_CTG _or ZFN_CAG _homodimer, respectively. Expression of the ZFNs in the HeLa/c-myc:(CTG)_n_•(CAG)_n _cell lines followed by PCR across the ectopic (CTG)_n_•(CAG)_n _TNRs demonstrated directly that hairpins form in vivo on both leading strand and lagging strand templates. Moreover, ZFN cleavage was inhibited in serum-deprived nondividing cells, implying that hairpin formation in this system is replication-dependent [7].

## Conclusions

(CTG)_n _and (CAG)_n _trinucleotide repeat sequences can form stable hairpins in vitro and in vivo, however, there is a facile transition of (CTG)_n _or (CAG)_n _hairpins to duplex in the presence of their complementary sequences in vitro. This suggests that other factors prolong the lifetime of (CTG)_n _and (CAG)_n _hairpins in vivo, among which may be MMR complexes, negative supercoiling behind replication or transcription forks, replication fork reversal, and protein, RNA, or leading strand binding of the hairpin complement. In HeLa/c-myc:(CTG)_n_•(CAG)_n _cells in culture, the rapid and efficient cleavage of hairpins in vivo by sequence- and structure-specific synthetic zinc finger nucleases, compared to the relatively extended time required before the appearance of expansions or contractions, raises another alternative, namely that hairpins are common but short-lived in vivo, and rarely result in TNR instability unless DNA replication or repair is perturbed. The efficient and accurate repair of preformed hairpins in cell extracts is consistent with this notion.

The instability of (CTG)_n_•(CAG)_n _repeats and the frequency of chromosome breakage are increased by mutations in yeast replisome proteins. These findings strengthen the link between replication fork instability, hairpin formation, the intra-S phase checkpoint, and DNA damage responses. The similar phenotypes of mutations in yeast replisome proteins and knockdown of orthologous human proteins suggest that evolutionarily conserved pathways operate to stabilize replication forks and maximize the integrity of replication.

Not surprisingly, cell cycle and checkpoint pathways appear to play a role in murine (CTG)_n_•(CAG)_n _stability. An outstanding difference between transgenic mouse systems and the human in vitro repair systems is the apparent contribution of the MMR proteins to instability in mice and the absence of their effect on in vitro repair. One possibility is that the preformation of stable hairpin substrates for in vitro repair may bypass an in vivo effect of chromatin structure, DNA metabolism, or MMR proteins.

Several fundamental questions concerning the mechanism of (CTG)_n_•(CAG)_n _instability remain to be addressed. For example, do contractions and expansions occur as consequences of the same process of replication, replication restart or postreplication repair? Do contractions and expansions occur in different phases of the mitotic cycle? Do contractions (or expansions) occur preferentially on the leading or lagging strand during replication? Are different pathways involved in the instability of various length TNRs? Does DNA damage promote hairpin formation? Which repair mechanisms are responsible for TNR instability in postmitotic cells? What is the mechanism of transcription-induced instability?

The use of yeast and transgenic mouse mutants, and RNAi to produce human cells and cell extracts deficient in specific functions promise to give insight into these questions, and thereby reveal second site genetic modifiers of TNR instability that can be used in prognosis and therapy.

## Competing interests

The authors declare that they have no competing interests.

## Authors' contributions

GL and ML drafted and revised the manuscript. Both authors read and approved the final manuscript.
